# Genomic and Gene-Expression Comparisons among Phage-Resistant Type-IV Pilus Mutants of *Pseudomonas syringae* pathovar *phaseolicola*

**DOI:** 10.1371/journal.pone.0144514

**Published:** 2015-12-15

**Authors:** Mark Sistrom, Derek Park, Heath E. O’Brien, Zheng Wang, David S. Guttman, Jeffrey P. Townsend, Paul E. Turner

**Affiliations:** 1 School of Natural Sciences, University of California Merced, Merced, 95343, CA, United States of America; 2 Department of Ecology and Evolutionary Biology, Yale University, New Haven, CT 06520, United States of America; 3 Department of Cell and Systems Biology, University of Toronto, Toronto, Ontario, M5S 3B2, Canada; 4 Centre for the Analysis of Genome Evolution & Function, University of Toronto, Ontario, M5S 3B2, Canada; 5 Department of Biostatistics, Yale School of Public Health, New Haven, CT 06520, United States of America; 6 Program in Computational Biology and Bioinformatics, Yale University, New Haven, CT 06520, United States of America; 7 Program in Microbiology, Yale University, New Haven, CT 06520, United States of America; University of the West of England, UNITED KINGDOM

## Abstract

*Pseudomonas syringae* pv. *phaseolicola* (Pph) is a significant bacterial pathogen of agricultural crops, and phage Φ6 and other members of the dsRNA virus family *Cystoviridae* undergo lytic (virulent) infection of Pph, using the type IV pilus as the initial site of cellular attachment. Despite the popularity of Pph/phage Φ6 as a model system in evolutionary biology, Pph resistance to phage Φ6 remains poorly characterized. To investigate differences between phage Φ6 resistant Pph strains, we examined genomic and gene expression variation among three bacterial genotypes that differ in the number of type IV pili expressed per cell: ordinary (wild-type), non-piliated, and super-piliated. Genome sequencing of non-piliated and super-piliated Pph identified few mutations that separate these genotypes from wild type Pph–and none present in genes known to be directly involved in type IV pilus expression. Expression analysis revealed that 81.1% of gene ontology (GO) terms up-regulated in the non-piliated strain were down-regulated in the super-piliated strain. This differential expression is particularly prevalent in genes associated with respiration—specifically genes in the tricarboxylic acid cycle (TCA) cycle, aerobic respiration, and acetyl-CoA metabolism. The expression patterns of the TCA pathway appear to be generally up and down-regulated, in non-piliated and super-piliated Pph respectively. As pilus retraction is mediated by an ATP motor, loss of retraction ability might lead to a lower energy draw on the bacterial cell, leading to a different energy balance than wild type. The lower metabolic rate of the super-piliated strain is potentially a result of its loss of ability to retract.

## Introduction

Halo blight is an economically significant disease of leguminous agricultural crops caused by the Gram-negative bacterial pathogen *Pseudomonas syringae* pv. *phaseolicola* (Pph) [1]. The genome of Pph comprises a 5.93 Mb chromosome and two plasmids, 132 kb and 52 kb in size [2]. Virulence in Pph requires a pathogenicity island located on the larger plasmid [3]. However, the ability for Pph and other Pseudomonads to attach to plants is via chromosomally-encoded type IV protein pili, which allow the bacterial cells to adhere to external structures (e.g., leaf surfaces) and to resist environmental perturbations such as rain splatter and wind, which could disrupt bacterial invasion of plant tissues [4–6]. One of the key mechanisms enabling motility of plant-pathogenic Pseudomonads—fundamental to their interactions with host plants—occurs through twitching motility [7], in which type IV pili are repeatedly extended and retracted to facilitate movement, analogous to grappling hooks. Spontaneous mutants of Pph that lack type IV pili show greatly reduced adherence to leaf surfaces and lower incidence of halo blight disease, whereas virulence of wildtype and pilus-deficient Pph mutants is equivalent when these strains are injected directly into bean leaves [1]. Therefore, adherence ability via type IV pili can be considered a conditional virulence factor directly involved in epiphytic colonization by *P*. *syringae* pathovars such as Pph.

Because type IV pili in Pseudomonads are critical for host plant attachment, it is unsurprising that some bacteriophages have evolved to exploit these conserved structures to facilitate viral infection of bacterial cells [8]. In particular, phage Φ6 and other members of the dsRNA virus family *Cystoviridae* undergo virulent (lytic) infection of Pph, using the type IV pilus as the initial site of cellular attachment [5,9]. Cystoviruses are characterized as having three dsRNA strands (segments) per particle and a lipid envelope. Attachment of phage Φ6 to the type IV pili of Pph is mediated by the viral attachment protein P3 [10,11]. Once attached, it is believed that retraction of the pilus moves one or more Φ6 particles in close proximity to the bacterial cell membrane, allowing membrane fusion [12] and ultimately phage entry into the cell via the viral lytic enzyme P5 [10,13]. Phage Φ6 then uses the host cell’s metabolism to cause a typical lytic infection, where cell lysis liberates mature virions capable of infecting host cells expressing the type IV pilus [9,14]. In addition to the popularity of Pph as a model for studying generalized plant-pathogen interactions [15], the infection dynamic between Pph and phage Φ6 can also be readily examined *in vitro*, facilitating studies of the evolution of host-parasite interactions [16–18].

Despite the growing popularity of Pph and phage Φ6 as a study system in evolutionary biology [19], the evolution of Pph resistance to phage Φ6 remains poorly characterized. Laboratory culture of Pph in the presence of phage Φ6 can be used to select for spontaneous bacterial mutants that are partially or fully resistant to phage infection [8,17]. We are unaware of any study that has characterized the full spectrum of mechanisms that allow Pph resistance to phage Φ6. However, prior *in vitro* experiments show that evolved resistance generally coincides with changes in the number of type IV pili produced by the bacteria [8,17], suggesting that type IV pili alteration may be the primary resistance mechanism. Notably, two of these resistant forms differ markedly in pili number: strains lacking type IV pili altogether, and strains with super-abundant type IV pili [8,17]. Resistance in non-piliated strains is due to the elimination of the initial attachment site for phage Φ6, although some low-level infection of these hosts occurs, presumably due to virus particles colliding directly with the cell surface [18]. Super-piliated host strains have elevated numbers of pili compared to wild type Pph, which suggests that these bacteria should be even more sensitive to phage Φ6 infection. Instead, super-piliated bacteria adsorb relatively larger numbers of phage particles as expected, but remain uninfected apparently because they do not retract their pili [8,17]; this inability to retract type IV pili appears similar to the mechanism described in *P*. *aeruginosa* [20]. Thus, super-piliated Pph strains ‘soak up’ virus particles in culture, but restrict phage infection because the non-retractable pili prevent virus particles from encountering the cell surface [17,18]. Wild-type, non-piliated and super-piliated Pph strains show similar maximal growth rates, stationary-phase densities and relative fitness abilities (i.e., competition for limited nutrients) in liquid culture in the laboratory [21]. More importantly, super-piliated mutants of Pph are similar to wild-type bacteria in terms of leaf adherence, and initiation of halo blight disease [1] This observation suggests that super-piliated phage-resistant mutants should be selectively favored over non-piliated (plant-adherence deficient) resistant mutants in the wild; however, we are unaware of any field studies that have directly compared the success of super-piliated versus non-piliated resistance mutants in natural populations of Pph, when exposed to phage attack.

To investigate differences between phage Φ6 resistant Pph strains and wild-type bacteria, we compared gene-expression profiles and whole-genome sequences of the two major types of resistant strains and wild-type bacteria. In particular, we examined genomic and gene expression variation between ordinary (wild-type) Pph and two spontaneous mutants of Pph that differed in the number of type IV pili expressed per cell: non-piliated, and super-piliated.

## Materials and Methods

### Strains

Strains were kindly provided by L. Chao (University of California, San Diego, La Jolla, CA), and are derived from a sample of wild-type (WT) Pph that was originally obtained from American Type Culture Collection (ATCC strain #21781, a.k.a. strain HB10Y). Both the non-piliated (P–) Pph strain and the super-piliated (P+) Pph strain were spontaneous mutants of WT Pph.

### Culture conditions

All strains were grown, diluted and plated at 25°C in Lysogeny broth as described in Dennehy et al. [18]. Cultures were initiated by placing a single colony grown on LB agar into a flask with 10 mL of LB medium. Flasks were shaken at 120 rpm with incubation for 24 hrs, which was sufficient for bacteria to achieve stationary-phase density (~1.65 × 10^9^ cells/ml).

### Genome sequencing and analysis

Sequencing followed the methods described in O'Brien et al. [22]. Briefly, DNA was isolated from 1 mL of stationary-phase culture using a Puregene Genomic DNA Purification Kit (Qiagen Canada, Toronto, ON) with the Gram-negative bacterial culture protocol with double volumes of each reagent, repeating the protein precipitation step twice, and spooling the DNA during the precipitation step. DNA was sheared to 200 base pairs (bp) using a Covaris S-series sample preparation system and paired-end sequencing libraries were prepared using sample preparation kits from Illumina (San Diego, CA). Libraries were multiplexed and run on a single lane of an Illumina GA IIx sequencer for 80 cycles per paired-end. Reads were mapped to the genome sequence of wild-type Pph strain HB10Y (Guttman unpublished data) using the CLC Genomics Workbench (Århus, Denmark), and polymorphisms were detected with the CLC Probabilistic Variant Caller. Annotations were copied from the fully sequenced reference strain Pph 1448A [2] using a Mauve whole genome alignment [23].

We evaluated mutational changes in five genes known to be directly involved in type IV pilus formation–type IV pilus assembly protein (PilM), type IV pilus biogenesis protein (PilN), type IV pilus biogenesis protein (PilO), type IV pilus biogenesis protein (PilP) and twitching motility protein (PilT) [5,24–26] in P+ and P- compared to wild type in order to asses any direct genotype–phenotype explanations for the observed phenotypic characteristics of these two strains.

For a well-characterized *P*. *syringae* pathovar distantly related to Pph (*P*. *syringae* pv. *tomato* strain #DC3000), putative sigma70 binding sites inferred from the RegulonDB database [27] were downloaded from http://www.ccg.unam.mx/Computational_Genomics/PromoterTools and homologous positions in Pph HB10Y were determined from a whole genome alignment made with Mauve [23]. Mutations occurring within 50bp upstream of the inferred transcription start sites were identified using BEDTools [28]. Pph HB10Y homologs were identified for 5121 out of 6716 putative sigma70 binding sites in *P*. *tomato* DC3000, and mutations were present within 50 bp of the putative transcription start sites for 8 (7 in the P+ strain, 1 in the P– strain).

### Gene expression analysis

Two biological replicates of each strain were cultured. For RNA extraction we used a standard Trizol approach. The bacterial cells (roughly 10^7^ to 10^8^ cells per ml of Trizol) were lysed in Trizol (Life Technology) and homogenized with a micropestle (Eppendorf), and RNAs were suspended in 0.1% diethylpyrocarbonate (DEPC) treated water. RNA quality was checked via spectrophotometry (NanoDrop). Reverse transcription of the RNA into cDNA was performed using the Invitrogen SuperScript Double-Stranded cDNA Synthesis kit (Thermo Fisher) using random primers. For each biological replicate, a replicate of the reverse transcription was performed using multi-targeted primers [29] designed for the Pph genome, instead of random primers, for first strand synthesis in order to increase yield. All cDNA samples were labeled using a NimbleGen One-Color (Cy3) DNA Labeling Kit (Madison, WI, USA). Hybridization of cDNA to NimbleGen 4 x 72k arrays (090326_P_syringe_B728a_expr; NimbleGen design ID #9510; Madison, WI, USA) according to the manufacturer guidelines was conducted by the Yale Center for Genome Analysis (New Haven, Connecticut, USA). Microarray slides were scanned with a GenePix 4000B (Axon Instruments, Foster City, CA). Spots were located and expression was quantified following NimbleGen Arrays User’s Guide, and spots with unusual morphology or with erratic signal intensity distribution were excluded from all analyses. Raw fluorescence data were normalized as in Townsend [30], and normalized data were then statistically analyzed using Bayesian Analysis of Gene Expression Levels (BAGEL, [30,31]). Genes were considered expressed and deemed well measured only when the median spot foreground exceeded the median background plus two standard deviations of the background intensity. Well measured genes were considered as significantly differentially expressed when P ≤ 0.05. Significantly up and down regulated genes with GO terms were used to determine significantly enriched GO terms using AmiGO 1.8 [32] with a significance level of 0.01 and a minimum of two genes for each enriched term. The JCVI CMR [33] database was used for background filtering and results were visualized using the AmiGO GO graph tool [32]. Visualization of gene expression differences among strains was done by using the clustergram function of the bioinformatics toolbox of the MatLab computational mathematics suite (Mathworks, Natick, MA, USA). The data were first mean-normalized and then transformed so that gene expression values were represented as standard deviations above or below the mean. Data were not sorted for statistical significance beforehand although later conclusions did take specific *P* values between strains into account.

## Results

### Strain isolation

The super-piliated and non-piliated mutants in the current study are spontaneous mutants of WT Pph strain HB10Y, obtained via laboratory assays that isolated spontaneous resistance mutants in bacterial populations subjected to phage Φ6 attack. This design facilitated genomic and gene-expression comparisons among strains, which may have been complicated if the strains were more distantly related, such as drawn from disparate natural sources and/or geographic locations. We noted that all three strains achieved similar stationary-phase densities (~1.65 × 10^9^ cells/ml) and maximal growth rates in Luria broth (Dennehy et al. 2007), indicating that average number of type IV pili expressed per cell constituted the major differences between WT Pph and each of the two spontaneous mutants.

### Genomics of super-piliated and non-piliated bacterial strains

Illumina-based genome sequencing using 80 base pair (bp) paired-end reads of the super-piliated (P+) and non-piliated (P–) strains produced 1.9 and 3.6 million reads respectively. Subsequent referenced alignment to the wildtype (WT) *P*. *syringae* pv. *phaseolicola* strain HB10Y resulted in a mean coverage of 14.0× and 24.4× for P+ and P–.

Alignment of the P+ strain identified 97 mutations separating it from WT (Table 1, S1 Table), consisting of 26 large-scale deletions, five deletion-insertion polymorphisms (DIPs), seven multi-nucleotide variants (MNVs) and 59 single nucleotide polymorphisms (SNPs). A notably large deletion of 1039 bp occurred in a retrotransposon hot spot (*rhs*) family gene, with the remainder averaging 120bp (54–269 bp). Fourteen of the deletions occurred in inter-genic regions, six in characterized genes and six in hypothetical proteins of unknown function. All five DIPs in P+ occurred in intergenic regions; three DIPs were in consecutive positions 158 bp downstream of the gene encoding the type III effector HopQ1. The remaining two DIPs were upstream of a prophage and fructose-1, 6-bisphosphate aldolase gene. Each observed MNV consisted of a 2–3bp change, six of which occurred in intergenic regions and the remaining MNV located in the *tolQ2* ORF, causing a threonine to leucine amino-acid substitution at the 121^st^ position. Of the 59 SNPs detected in P+, 15 occurred in ORFs, of which six were non-synonymous amino acid changes.

**Table 1:** A summary of genomic and gene expression differences between super-piliated and non-piliated Pph when compared to WT.

Mutation Type	Strain	
	P+	P–
Large Scale deletions	26	4
Deletion-Insertion Polymorphisms	5	8
Multi-nucleotide Variants	7	8
Single Nucleotide Polymorphisms	59	62
Total	97	82
Expression Differences		
Genes UP–regulated	503	104
Genes Down-regulated	451	28
Gene Ontology Terms UP–regulated	4	3
Gene Ontology Terms Down-regulated	38	1

Alignment of the P– strain identified 82 total mutations separating it from WT (Table 1, S2 Table) consisting of four large-scale deletions, eight DIPs, eight MNVs, and 62 SNPs. A single large deletion of 2116 bp occurred in the gene encoding the *rulA* protein, with the remainder averaging 80 bp (53–112 bp). Four of the eight observed DIPs occurred in a GntR family transcriptional regulator, causing frameshift mutations at amino acids 131 and 132. Two DIPs occurred in a putative H+ antiporter subunit C causing a frameshift at Phe_50_, and one DIP was present in the *PilX* ORF causing a frameshift mutation at Ile_192_. The remaining DIP was present in an intergenic region. The eight observed MNVs ranged from 2–5bp in length. Five of the eight MNVs were in intergenic regions. However, the remaining three MNVs were located within the H+ antiporter, a GntR family transcriptional regulator and a TolQ protein causing frameshifts at Phe_50_, at Asp_131_ and an amino acid change from Thr_121_ to Leu, respectively. Of the 62 SNPs observed in P–, 34 were located in intergenic regions, and of the 28 SNPs occurring in confirmed ORFs, 16 represented non-synonymous changes.

None of the observed mutations were present in any of the genes known to be directly involved in type IV pilus formation (i.e., PilM, PilN, PilO, PilP and PilT) [5,24–26] in P+ or P-.

Putative phage Φ6 binding site mutations are shown in Table 2. One was detected in P–, with seven detected in P+. The single binding site mutation in P– represents a SNP (G to C) upstream of a citrate transporter gene, and downstream of a hypothetical protein. The binding site mutations in P+ include an MNV upstream of type III effector *HopQ2* which was also significantly upregulated in comparison with WT, along with six deletions ranging in size from 64–269 bp. Of the genes potentially affected by these deletions, a *LuxR* family D—binding response regulator and an iron (III) dicitrate transport protein *FecA* were significantly downregulated in P+, and a ribosomal subunit interface protein, a periplasmic amino acid-binding protein, and a hypothetical protein were significantly upregulated in P+.

**Table 2:** Details putative sigma70 binding sites inferred from the RegulonDB database for which significant regulatory changes were detected.

Reference Position	Consensus Position	Variation Type	Length	Reference	Allele Variations	Overlapping Annotations	Upstream annotation	Upstream distance	Upstream orientation	Downstream annotation	Downstream distance	Downstream orientation
4059	4059	MNV	3	—-	GCT		PSPPH_A0012/hopQ1 (type III effector HopQ1)	158	−1			
546554	546628	Deletion	75				PSPPH_0642 (LuxR family D—binding response regulator)	53	1	"PSPPH_0643 (polyamine ABC transporter, ATP-binding protein)"	197	1
101598	101722	Deletion	125				"PSPPH_1090 (ribosomal subunit interface protein, putative)"	135	−1	PSPPH_1091 (iron(III) dicitrate transport protein fecA)	122	–1
272415	272478	Deletion	64				"PSPPH_1387 (amino acid ABC transporter, permease protein)"	12	−1	"PSPPH_1388 (amino acid ABC transporter, periplasmic amino acid-binding protein)"	6	–1
141653	141921	Deletion	269			PSPPH_4466 (hypothetical protein)						
259583	259844	Deletion	262			PSPPH_0408 (hypothetical protein)						
2	127	Deletion	126			PSPPH_4902 (molybdenum-pterin binding domain-containing protein)						
14090	14090	SNP	1	G	C		PSPPH_0186 (citrate transporter)	159	−1	PSPPH_0187 (hypothetical protein)	136	1

### Transcriptome Analysis

**Fig 1:**
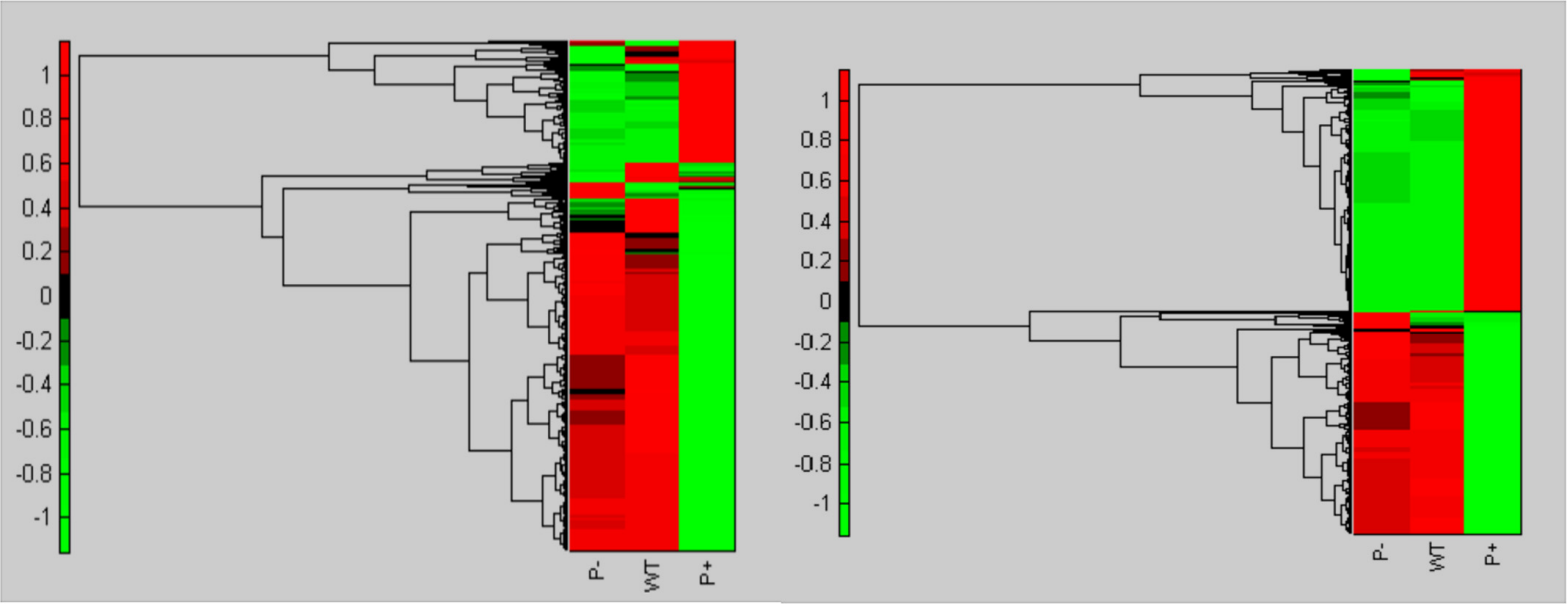
Clustergram of standardized values of all genes (left) and significantly (*P* < 0.05) differentially expressed genes (right). Significantly differentially expressed genes are those that are significantly up-regulated (red) /down-regulated (green) in P+ or P– relative to WT.

The gene expression (GE) profiles of the 3 strains were compared using a clustergram and heatmap (Fig 1) analysis applied to all well measured genes (*N* = 5069) which comprised approximately 98% of the total annotated genes in the Pph genome, and applied to genes that showed a significant difference (*P* < 0.05) between strains (*N* = 1009). Both analyses showed that the GE profile of P+ differed more substantially from WT than did P– (Fig 1) with higher significance when restricted to significantly different genes. Two major clusters of genes were observed: one cluster contained genes highly expressed in P+ compared to WT and P–, and another contained genes highly expressed in WT and P– in comparison with P+.

**Fig 2:**
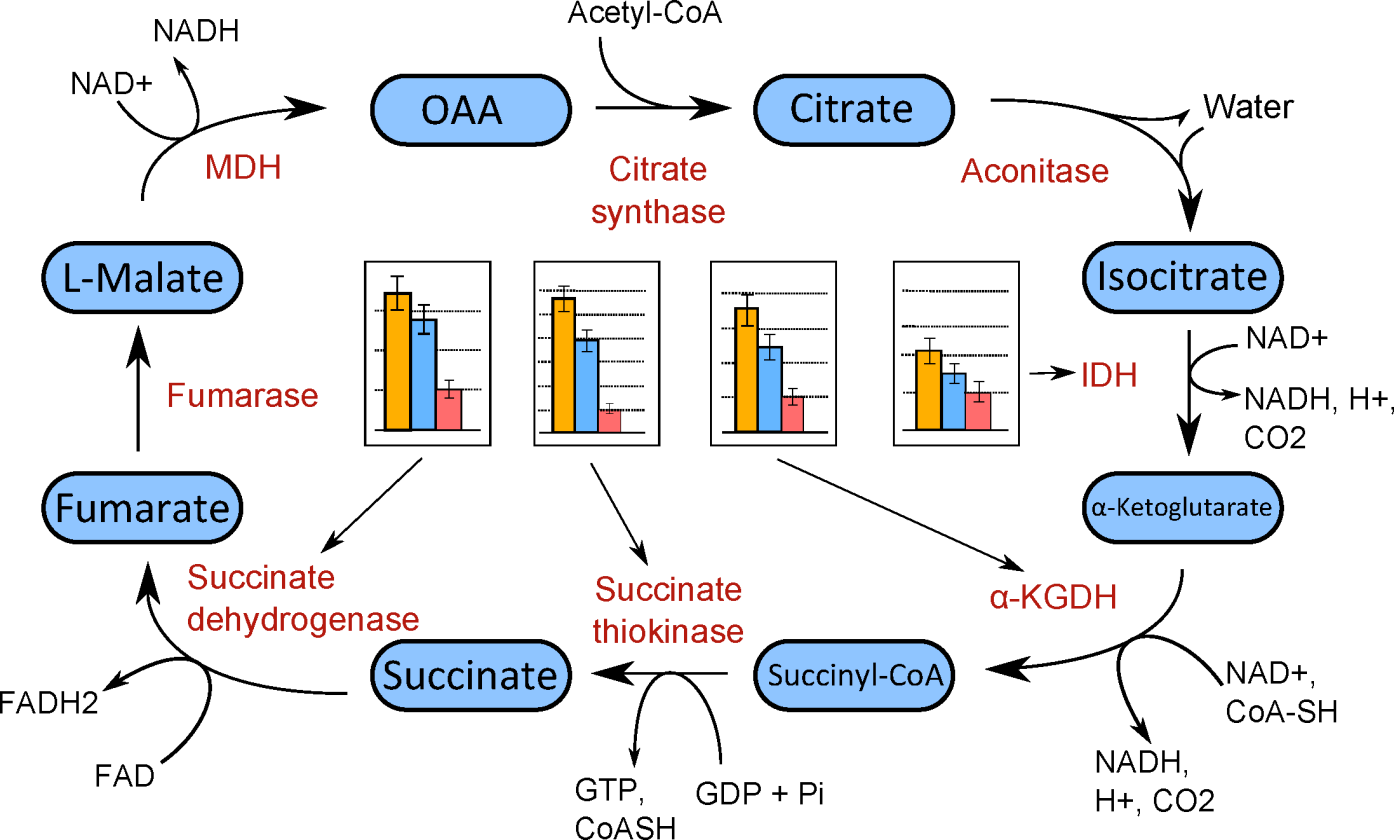
Expression levels of key enzymes of the TCA cycle in P– (yellow), WT (blue), and P+ (red) strains.

Of the 5069 genes measured on the microarray, 954 genes (18.8%) showed significant expression-level differences between WT and P+, with 503 genes upregulated and 451 genes downregulated (Fig 2, Table 1, S3 Table). To further examine genome-wide differences in GE, genes upregulated in P+ were analyzed for GO term enrichment (S1 Fig). We observed that 10 terms were significantly enriched, all of which pertained to processes of transposition and DNA metabolism. An enrichment analysis of genes down-regulated in P+ showed significant enrichment for 48 different GO terms. Visualizing the organization of these GO terms revealed that a few key biological processes were enriched: gene expression and translation, nucleotide and ribonucleotide biosynthesis, and cellular respiration (S2 Fig). Of the 5069 genes measured on the microarray, 132 (2.6%) genes significantly differed in gene expression levels between WT and P–. Of these 132 genes, 104 genes were up-regulated and 28 genes were down-regulated (S1 Table). We found that 49 GO terms were enriched for P– up-regulation and 31 GO terms were enriched for down-regulation. Visualization of these GO terms showed particularly high enrichment for intracellular organelles and ribosomal structure in genes up-regulated in P– (S3 Fig), and genes associated with conjugation as well as protein transport and secretion showed high levels of down-regulation in P– (S4 Fig).

## Discussion

### Genomic differences between P+ and P– phage resistant P. syringae pv. phaseolicola

Genome sequencing of P+ and P– strains identified 97 and 82 mutations that differed from WT strain, respectively. These mutations consisted of 26 deletions, five DIPs, seven MNVs and 59 single SNPs in P+, and four deletions, eight DIPs, eight MNVs, and 62 SNPs in P– (Table 1). Of these differences, 20 and 27 mutations resulted in non-synonymous changes to protein coding genes in P+ and P– strains. These observations constitute a relatively very small number of protein coding changes—approximately one mutation per 300 kb and 220 kb respectively, indicating that the observed differences in pilus number associated with P+ and P– stem from a small number of large effect mutational changes. Genes affected by protein coding changes in P+ included SNPs located in a general virulence surface antigen protein, and a type III effector AvrB4-2—both loci are associated with plant-disease virulence in Pph [15]. Genes affected by protein coding changes in P– were a large deletion in the RulA protein, which confers UV resistance in Pph [34]. Both P+ and P– had MNVs in the *tolQ2* gene—including identical mutations from threonine to leucine at the 121^st^ position. *tolQRA* genes encode membrane proteins that determine phage sensitivity versus resistance in *Escherichia coli*, where *tolQRA* mutants were resistant to phage infection [35]. It is therefore possible that mutations in TolQ membrane proteins play a role in both entry of phage Φ6 into Pph cells, as well as resistance of Pph to phage Φ6 attack. The specifics of TolQ mutations associated with phage resistance deserve further investigation.

Interestingly, we did not find any mutations in genes known to be required for pilus expression (i.e. *pilM*, *pilN*, *pilO*, *pilP* and *pilT*) [5,24–26]. This indicates that the link between the P+ and P- phenotypes and their corresponding genotypes is likely to be complex and possibly regulatory in nature. However, one possibility not explored by the current study is that the pertinent mutations occur on plasmids.

### Interactions between mutation and gene expression

Of the 20 non-synonymous mutations in P+, six were located in genes with significant expression differences compared to WT; four were up-regulated and two were down-regulated. Of the four genes which were up-regulated, two were annotated as hypothetical proteins of unknown function. However, the two remaining upregulated genes were related to Pph virulence. The first is AvrB4-2, a type III effector protein associated with Pph virulence in common bean (*Phaseolus vulgaris*) plants [36]. P+ had a SNP mutation in *AvrB4-2* that caused an amino acid change from aspartic acid to asparagine at the 240^th^ position. *Avr* genes in *P*. *syringae* are involved in host-specific resistance mechanisms between the bacterium and its plant hosts [37]. The second SNP was initially identified in a search of 19 *P*. *syringae* genome sequences for genes related to bacterial pathogenicity [38]. A valine at the third amino acid position of the gene was changed to a leucine.

In P–, only one of the 26 genes containing non-synonymous mutations showed a significant expression difference compared to WT: a phage integrase-family recombinase. In this gene, two mutations were observed: a change from leucine to proline at position 216, and a change from methionine to leucine at position 219. Also of interest is the combination of a binding site mutation and up-regulation of a type III effector *hopQ2* gene in P–. Type III virulence effector proteins are important for *P*. *syringae* to infect and grow in plant hosts, and thus these mutations may yield insights into the virulence of P– strains of *P*. *syringae* (i.e., aside from the expected reduced conditional virulence on plant hosts, due to the lack of type IV pili in P–strains).

### Gene expression differences between P+ and P– phage resistant P. syringae pv. phaseolicola

GE Profiling demonstrated that P+ had significantly more genes both up and down-regulated in comparison to WT than P–: compared to WT, 503 and 104 genes were significantly up-regulated and 451 and 28 genes were downregulated in P+ and P–. GO term enrichment analysis revealed four and 40 GO terms enriched for up-regulation in P+ and P–, and 38 and one GO terms enriched for down-regulation in P+ and P–.

The exceptionally low number of GO terms enriched for up-regulation in P+ suggests that transcription and DNA metabolism are likely to be key processes in P+ resistance to phage infection, and the genes annotated for each term represent candidates for further, gene specific analysis. Down-regulation of genes associated with gene expression and translation, nucleotide and ribonucleotide biosynthesis, and cellular respiration in P+ (Fig 2) indicates the potential importance of these pathways in P+ resistance to phage infection.

The up-regulation of genes involved in gene expression and ribosome production in P–suggests the up-regulation of protein production in general. Additionally, the up-regulation of genes associated with cellular respiration and ATP synthesis indicates that energy production in general increased in P–. The observed down-regulation of pili-related genes in P– is unsurprising given that this strain lacks pili, however the specific functions of the proteins within this down-regulated set of genes have yet to be investigated.

Perhaps of most interest is the observation that 81.1% of GO terms up-regulated in P– were shown to be down-regulated in P+ (Fig 1, S3 Table). This result indicates that regulatory changes in similar pathways have led to divergent, independent mechanisms of phage resistance in Pph. A number of genes associated with respiration–specifically TCA cycle, aerobic respiration and acetyl-CoA metabolism genes–were up-regulated in P– and downregulated in P+.

There were 10 and seven genes annotated for all three of these GO terms differentially expressed in comparison with WT in P+ and P– respectively, of which four were common to both. The four genes were succinate dehydrogenase, alpha-ketoglutarate dehydrogenase, isocitrate dehydrogenase, and succinyl-CoA synthetase–all encoding key enzymes in the TCA cycle. Plotting the expression levels of these genes reveals a similar pattern of greatest expression in P– and lowest expression in P+ (Fig 2). Viewing these genes in the context of their location in the TCA cycle (Fig 2) reveals that the enzymes they encode are responsible for catalyzing energy-yielding reactions, either in terms of a nucleoside triphosphate or a reduced cofactor. In general, kinetic and allosteric feedback loops between metabolites and enzymes in a biological pathway generally maintain a degree of stability, and the increases or reductions in the activity of one gene versus another does not significantly change flux through the pathway. However, if all of the enzymes are up-regulated/down-regulated in concert, such as in the case of P+ and P–, the whole pathway would be affected. Thus, the expression pattern of these four genes suggests that the TCA pathway is generally up-regulated/down-regulated in P+ and P– respectively, and that its pathway flux relates to pilus regulation

The down-regulation of the TCA pathway could indicate lower metabolic rate of the P+ strain, potentially a result of its loss of retraction ability. Pilus retraction is mediated by an ATP motor, so loss of retraction ability could lead to a draw of ATP on the bacterial system [39]. These conclusions are speculative, however, and require further experiments for confirmation.

The divergent expression patterns of these TCA cycle genes in P– versus P+ reflect the general reciprocal GE profiles of the two strains. Overall, genes up-regulated in P– are down-regulated in P+ and vice versa (S3 Table). The only exception to these reciprocal GE profiles is that transposition is up-regulated in both P– and P+. The fact that in P– the transposition genes are up-regulated, while pili genes are down-regulated may suggest a mutation causing a fundamental disconnect between these two otherwise co-regulated processes. This observation will be a continued area of study, since the exact regulatory mechanisms behind transposition in Pph are not well known.

The results of our study are useful for general research efforts harnessing phage Φ6 and Pph. In particular, these microbial strains have been used in prior studies in experimental ecology and evolutionary biology, especially to conduct experimental evolution that addressed fundamental questions in these fields [13,16,40,41]. However, these prior studies have not focused on co-evolutionary interactions between phage Φ6 and Pph, perhaps because resulting genetic changes in the host bacteria could not be compared to existing full genome sequences of the founding strains. Our study addresses this limitation and lends more power to the phage Φ6/Pph model system, because such work can now harness affordable re-sequencing approaches to compare ancestral and derived strains of bacteria. Also, recent studies show that Cystoviruses are readily found in the terrestrial phyllosphere in temperate environments, especially in association with bacteria residing on or within common legumes such as white clover plants [42–45]. These studies emphasize the prevalence of RNA phages in terrestrial biomes [42–45], and to our knowledge Cystoviruses constitute the only non-marine RNA phages that are actively researched in the wild [46]. Whereas these earlier studies were used to infer population structure and isolation-by-distance in Cystoviruses, our study is useful for future empirical work that manipulates Cystoviruses and Pph strains on legumes in field experiments or in the greenhouse. For example, the bacteria characterized in our study could be used to examine the effects of phage infection on the community dynamics of bacteria that differ in plant pathogenicity, under realistic conditions such as on leaf surfaces. Overall, characterization of Pph pili mutants in the current study support the crucial need to further develop emerging models in phage/bacteria interactions, which should broaden the understanding of symbiotic interactions between the most prevalent entities in the biosphere [47,48].

## Supporting Information

S1 Fig Visualization of the GO terms enriched in the genes up-regulated in P+. Shading corresponds to level of significance. The vertical axis generally indicates greater or lesser specificity of the GO term because of the hierarchical definitions of the terms. The three major biological functions that are enriched are those of Gene expression and translation, nucleotide and ribonucelotide biosynthesis, and respiration. The majority of unboxed terms are mostly general terms lacking a unifying biological function.
(PDF)

S2 Fig Visualization of the GO terms enriched in the genes down-regulated in P+. Shading corresponds to level of significance. The vertical axis generally indicates greater or lesser specificity of the GO term because of the hierarchical definitions of the terms. The three major biological functions that are enriched are those of gene expression and translation, nucleotide and ribonucelotide biosynthesis, and respiration. The majority of unboxed terms are mostly general terms lacking a unifying biological function.
(PDF)

S3 Fig Visualization of the GO terms enriched in the genes upregulated in P–. Shading corresponds to level of significance. The vertical axis generally indicates greater or lesser specificity of the GO term because of the hierarchical definitions of the terms. The three major biological functions that are enriched are those of gene expression and translation, nucleotide and ribonucelotide biosynthesis, and respiration. The majority of unboxed terms are mostly general terms lacking a unifying biological function.
(PDF)

S4 Fig Visualization of the GO terms enriched in the genes downregulated in P–. Shading corresponds to level of significance. The vertical axis generally indicates greater or lesser specificity of the GO term because of the hierarchical definitions of the terms. The three major biological functions that are enriched are those of gene expression and translation, nucleotide and ribonucelotide biosynthesis, and respiration. The majority of unboxed terms are mostly general terms lacking a unifying biological function.
(PDF)

S1 Table Genomic mutations present in super-piliated Pph in comparison to WT. (XLSX)

S2 Table Genomic mutations present in non-piliated Pph in comparison to WT. (XLSX)

S3 Table Gene expression differences between super and non-piliated Pph in comparison to WT. The number 1 indicates genes which are significantly up or down regulated, 0 indicates genes which do not show a significant difference.
(XLSX)

## References

[1] Romantschuk M, Bamford DH. The causal agent of halo blight in bean, Pseudomonas syringae pv. phaseolicola, attaches to stomata via its pili. Microb Pathog. 1986;1: 139–148. 10.1016/0882-4010(86)90016-1 2907769

[2] Joardar V, Lindeberg M, Jackson RW, Selengut J, Dodson R, Brinkac LM, et al Whole-genome sequence analysis of Pseudomonas syringae pv. phaseolicola 1448A reveals divergence among pathovars in genes involved in virulence and transposition. J Bacteriol. 2005;187: 6488–6498. 10.1128/JB.187.18.6488-6498.2005 16159782 PMC1236638

[3] Jackson RW, Athanassopoulos E, Tsiamis G, Mansfield JW, Sesma A, Arnold DL, et al Identification of a pathogenicity island, which contains genes for virulence and avirulence, on a large native plasmid in the bean pathogen Pseudomonas syringae pathovar phaseolicola. Proc Natl Acad Sci U S A. 1999;96: 10875–10880. 10.1073/pnas.96.19.10875 10485919 PMC17976

[4] Romantschuk M, Nurmiaho-Lassila E-L, Roine E, Suoniemi A. Pilus-mediated adsorption of Pseudomonas syringae to the surface of host and non-host plant leaves. Journal of General Microbiology. 1993 pp. 2251–2260. 10.1099/00221287-139-9-2251

[5] Roine E, Nunn DN, Paulin L, Romantschuk M. Characterization of genes required for pilus expression in Pseudomonas syringae pathovar phaseolicola. J Bacteriol. 1996;178: 410–417. 8550460 10.1128/jb.178.2.410-417.1996 PMC177672

[6] Burdman S, Bahar O, Parker JK, de la Fuente L. Involvement of type IV pili in pathogenicity of plant pathogenic bacteria. Genes (Basel). 2011;2: 706–735. 10.3390/genes2040706 24710288 PMC3927602

[7] Mattick JS. Type IV pili and twitching motility. Annu Rev Microbiol. 2002;56: 289–314. 10.1146/annurev.micro.56.012302.160938 12142488

[8] Romantschuk M, Bamford DH. Function of Pili in Bacteriophage-Phi-6 Penetration. J Gen Virol. 1985;66: 2461–2469. Available: http://eutils.ncbi.nlm.nih.gov/entrez/eutils/elink.fcgi?dbfrom=pubmed&id=2865329&retmode=ref&cmd=prlinks 2865329 10.1099/0022-1317-66-11-2461

[9] Mindich L. Phages with segmented double-stranded RNA genomes In: R. C, ST A, editors. The Bacteriophages. New York: Oxford University Press; 2006 pp. 197–210.

[10] Gottlieb P, Metzger S, Romantschuk M, Carton J, Strassman J, Bamford DH, et al Nucleotide sequence of the middle dsRNA segment of bacteriophage phi 6: placement of the genes of membrane-associated proteins. Virology. 1988;163: 183–190. 10.1016/0042-6822(88)90245-0 3347997

[11] Duffy S, Turner PE, Burch CL. Pleiotropic costs of niche expansion in the RNA bacteriophage Φ6. Genetics. 2006;172: 751–757. 10.1534/genetics.105.051136 16299384 PMC1456241

[12] Bamford DH, Romantschuk M, Somerharju PJ. Membrane fusion in prokaryotes: bacteriophage phi 6 membrane fuses with the Pseudomonas syringae outer membrane. EMBO J. 1987;6: 1467–1473. 3608985 10.1002/j.1460-2075.1987.tb02388.x PMC553953

[13] Dessau M, Goldhill D, McBride RL, Turner PE, Modis Y. Selective Pressure Causes an RNA Virus to Trade Reproductive Fitness for Increased Structural and Thermal Stability of a Viral Enzyme. PLoS Genet. 2012;8 10.1371/journal.pgen.1003102 PMC3510033 23209446

[14] Bamford DH, Palva ET, Lounatmaa K. Ultrastructure and life cycle of the lipid-containing bacteriophage phi 6. J Gen Virol. 1976;32: 249–259. 10.1099/0022-1317-32-2-249 798023

[15] Arnold DL, Lovell HC, Jackson RW, Mansfield JW. Pseudomonas syringae pv. phaseolicola: From “has bean” to supermodel. Mol Plant Pathol. 2011;12: 617–627. 10.1111/j.1364-3703.2010.00697.x 21726364 PMC6640400

[16] Turner PE, Chao L. Prisoner’s dilemma in an RNA virus. Nature. 1999;398: 441–443. 10.1038/18913 10201376

[17] Lythgoe KA, Chao L. Mechanisms of coexistence of a bacteria and a bacteriophage in a spatially homogeneous environment. Ecol Lett. 2003;6: 326–334. 10.1046/j.1461-0248.2003.00433.x

[18] Dennehy JJ, Abedon ST, Turner PE. Host density impacts relative fitness of bacteriophage Φ6 genotypes in structured habitats. Evolution (N Y). 2007;61: 2516–2527. 10.1111/j.1558-5646.2007.00205.x 17725627

[19] Dennehy JJ. Bacteriophages as model organisms for virus emergence research. Trends in Microbiology. 2009 pp. 450–457. 10.1016/j.tim.2009.07.006 19765997 PMC7127698

[20] Johnson K, Lory S. Characterization of Pseudomonas aeruginosa mutants with altered piliation. J Bacteriol. 1987;169: 5663–7. Available: http://www.pubmedcentral.nih.gov/articlerender.fcgi?artid=214031&tool=pmcentrez&rendertype=abstract 2445731 10.1128/jb.169.12.5663-5667.1987 PMC214031

[21] Dennehy JJ, Friedenberg NA, Yang YW, Turner PE. Virus population extinction via ecological traps. Ecol Lett. 2007;10: 230–240. 10.1111/j.1461-0248.2006.01013.x 17305806

[22] O’Brien HE, Gong Y, Fung P, Wang PW, Guttman DS. Use of low-coverage, large-insert, short-read data for rapid and accurate generation of enhanced-quality draft Pseudomonas genome sequences. PLoS One. 2011;6 10.1371/journal.pone.0027199 PMC3206934 22073286

[23] Darling ACE, Mau B, Blattner FR, Perna NT. Mauve: Multiple alignment of conserved genomic sequence with rearrangements. Genome Res. 2004;14: 1394–1403. 10.1101/gr.2289704 15231754 PMC442156

[24] Roine E, Raineri DM, Romantschuk M, Wilson M, Nunn DN. Characterization of type IV pilus genes in Pseudomonas syringae pv. tomato DC3000. Mol Plant Microbe Interact. 1998;11: 1048–1056. 10.1094/MPMI.1998.11.11.1048 9805392

[25] Taguchi F, Ichinose Y. Role of type IV pili in virulence of Pseudomonas syringae pv. tabaci 6605: correlation of motility, multidrug resistance, and HR-inducing activity on a nonhost plant. Mol Plant Microbe Interact. 2011;24: 1001–1011. 10.1094/MPMI-02-11-0026 21615203

[26] Nguyen LC, Taguchi F, Tran QM, Naito K, Yamamoto M, Ohnishi-Kameyama M, et al Type IV pilin is glycosylated in Pseudomonas syringae pv. tabaci 6605 and is required for surface motility and virulence. Mol Plant Pathol. 2012;13: 764–774. 10.1111/j.1364-3703.2012.00789.x 22353307 PMC6638785

[27] Huerta AM, Salgado H, Thieffry D, Collado-Vides J. RegulonDB: a database on transcriptional regulation in Escherichia coli. Nucleic Acids Res. 1998;26: 55–59. 9399800 10.1093/nar/26.1.55 PMC147189

[28] Quinlan AR, Hall IM. BEDTools: A flexible suite of utilities for comparing genomic features. Bioinformatics. 2010;26: 841–842. 10.1093/bioinformatics/btq033 20110278 PMC2832824

[29] Adomas AB, Lopez-Giraldez F, Clark TA, Wang Z, Townsend JP. Multi-targeted priming for genome-wide gene expression assays. BMC Genomics. 2010;11: 477 10.1186/1471-2164-11-477 20716356 PMC3091673

[30] Townsend JP. Resolution of large and small differences in gene expression using models for the Bayesian analysis of gene expression levels and spotted DNA microarrays. BMC Bioinformatics. 2004;5: 54 10.1186/1471-2105-5-54 15128431 PMC420235

[31] Townsend JP, Hartl DL. Bayesian analysis of gene expression levels: statistical quantification of relative mRNA level across multiple strains or treatments. Genome Biol. 2002;3: RESEARCH0071 10.1186/gb-2002-3-12-research0071 12537560 PMC151173

[32] Carbon S, Ireland A, Mungall CJ, Shu S, Marshall B, Lewis S, et al AmiGO: Online access to ontology and annotation data. Bioinformatics. 2009;25: 288–289. 10.1093/bioinformatics/btn615 19033274 PMC2639003

[33] Davidsen T, Beck E, Ganapathy A, Montgomery R, Zafar N, Yang Q, et al The comprehensive microbial resource. Nucleic Acids Res. 2009;38 10.1093/nar/gkp912 PMC2808947 19892825

[34] Sundin GW, Jacobs JL, Murillo J. Sequence diversity of rulA among natural isolates of Pseudomonas syringae and effect on function of rulAB-mediated UV radiation tolerance. Appl Environ Microbiol. 2000;66: 5167–5173. 10.1128/AEM.66.12.5167-5173.2000 11097885 PMC92439

[35] Picken RN, Beacham IR. Bacteriophage-resistant mutants of Escherichia coli K12. Location of receptors within the lipopolysaccharide. J Gen Microbiol. 1977;102: 305–318. 10.1099/00221287-102-2-305 336845

[36] Zumaquero A, Macho AP, Rufián JS, Beuzón CR. Analysis of the role of the type III effector inventory of Pseudomonas syringae pv. phaseolicola 1448a in interaction with the plant. J Bacteriol. 2010;192: 4474–4488. 10.1128/JB.00260-10 20601478 PMC2937392

[37] Lee CC, Wood MD, Ng K, Andersen CB, Liu Y, Luginbühl P, et al Crystal structure of the type III effector AvrB from Pseudomonas syringae. Structure. 2004;12: 487–494. 10.1016/j.str.2004.02.013 15016364

[38] Baltrus DA, Nishimura MT, Romanchuk A, Chang JH, Mukhtar MS, Cherkis K, et al Dynamic evolution of pathogenicity revealed by sequencing and comparative genomics of 19 pseudomonas syringae isolates. PLoS Pathog. 2011;7 10.1371/journal.ppat.1002132 PMC3136466 21799664

[39] Helaine S, Dyer DH, Nassif X, Pelicic V, Forest KT. 3D structure/function analysis of PilX reveals how minor pilins can modulate the virulence properties of type IV pili. Proc Natl Acad Sci U S A. 2007;104: 15888–15893. 10.1073/pnas.0707581104 17893339 PMC2000383

[40] Goldhill DH, Turner PE. The evolution of life history trade-offs in viruses. Current Opinion in Virology. 2014 pp. 79–84. 10.1016/j.coviro.2014.07.005 25087040

[41] Montville R, Froissart R, Remold SK, Tenaillon O, Turner PE. Evolution of mutational robustness in an RNA virus. PLoS Biol. 2005;3: 1939–1945. 10.1371/journal.pbio.0030381 PMC1275523 16248678

[42] Mindich L, Qiao X, Qiao J, Onodera S, Romantschuk M, Hoogstraten D. Isolation of additional bacteriophages with genomes of segmented double- stranded RNA. J Bacteriol. 1999;181: 4505–4508. 10419946 10.1128/jb.181.15.4505-4508.1999 PMC103579

[43] Silander OK, Weinreich DM, Wright KM, O’Keefe KJ, Rang CU, Turner PE, et al Widespread genetic exchange among terrestrial bacteriophages. Proc Natl Acad Sci U S A. 2005;102: 19009–19014. 10.1073/pnas.0503074102 16365305 PMC1323146

[44] O’Keefe KJ, Silander OK, McCreery H, Weinreich DM, Wright KM, Chao L, et al Geographic differences in sexual reassortment in RNA phage. Evolution (N Y). 2010;64: 3010–3023. 10.1111/j.1558-5646.2010.01040.x 20500219

[45] Díaz-Muñoz SL, Tenaillon O, Goldhill D, Brao K, Turner PE, Chao L. Electrophoretic mobility confirms reassortment bias among geographic isolates of segmented RNA phages. BMC Evol Biol. 2013;13: 206 10.1186/1471-2148-13-206 24059872 PMC3848951

[46] Culley AI, Lang AS, Suttle CA. Metagenomic analysis of coastal RNA virus communities. Science. 2006;312: 1795–1798. 10.1126/science.1127404 16794078

[47] Hendrix RW. Bacteriophages: evolution of the majority. Theor Popul Biol. 2002;61: 471–480. 10.1006/tpbi.2002.1590 12167366

[48] Wasik BR, Turner PE. On the biological success of viruses. Annu Rev Microbiol. 2013;67: 519–41. 10.1146/annurev-micro-090110-102833 23808330

