# Supplementary figures and images for: Genomic and Gene-Expression Comparisons among Phage-Resistant Type-IV Pilus Mutants of Pseudomonas syringae pathovar phaseolicola
Source: PLoS One. 2015 Dec 15;10(12):e0144514. doi: 10.1371/journal.pone.0144514 (PMC4687649; doi:10.1371/journal.pone.0144514)

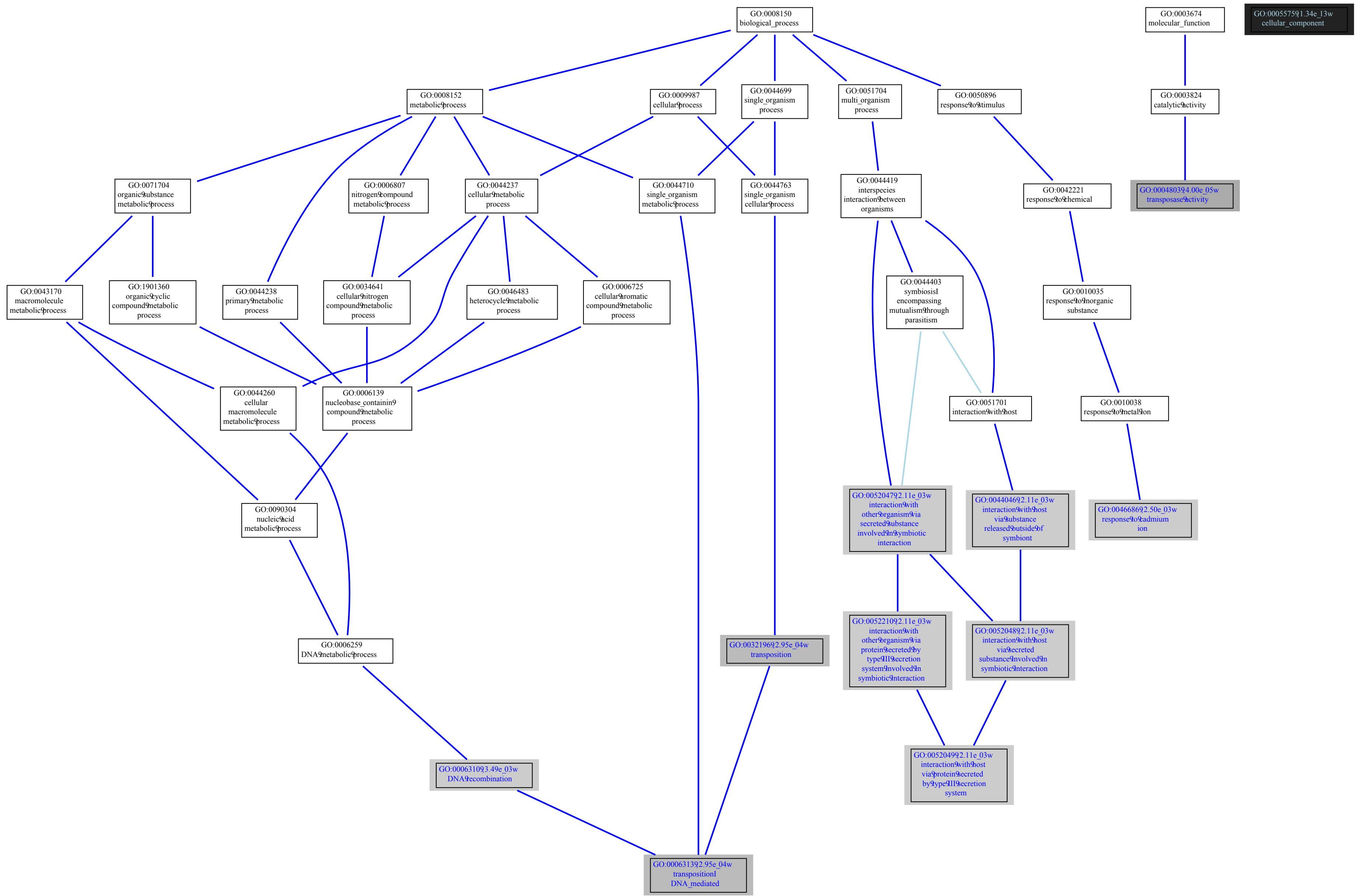

Supplement: S1 Fig — Shading corresponds to level of significance. The vertical axis generally indicates greater or lesser specificity of the GO term because of the hierarchical definitions of the terms. The three major biological functions that are enriched are those of Gene expression and translation, nucleotide and ribonucelotide biosynthesis, and respiration. The majority of unboxed terms are mostly general terms lacking a unifying biological function. (PDF) [file pone.0144514.s001.pdf]

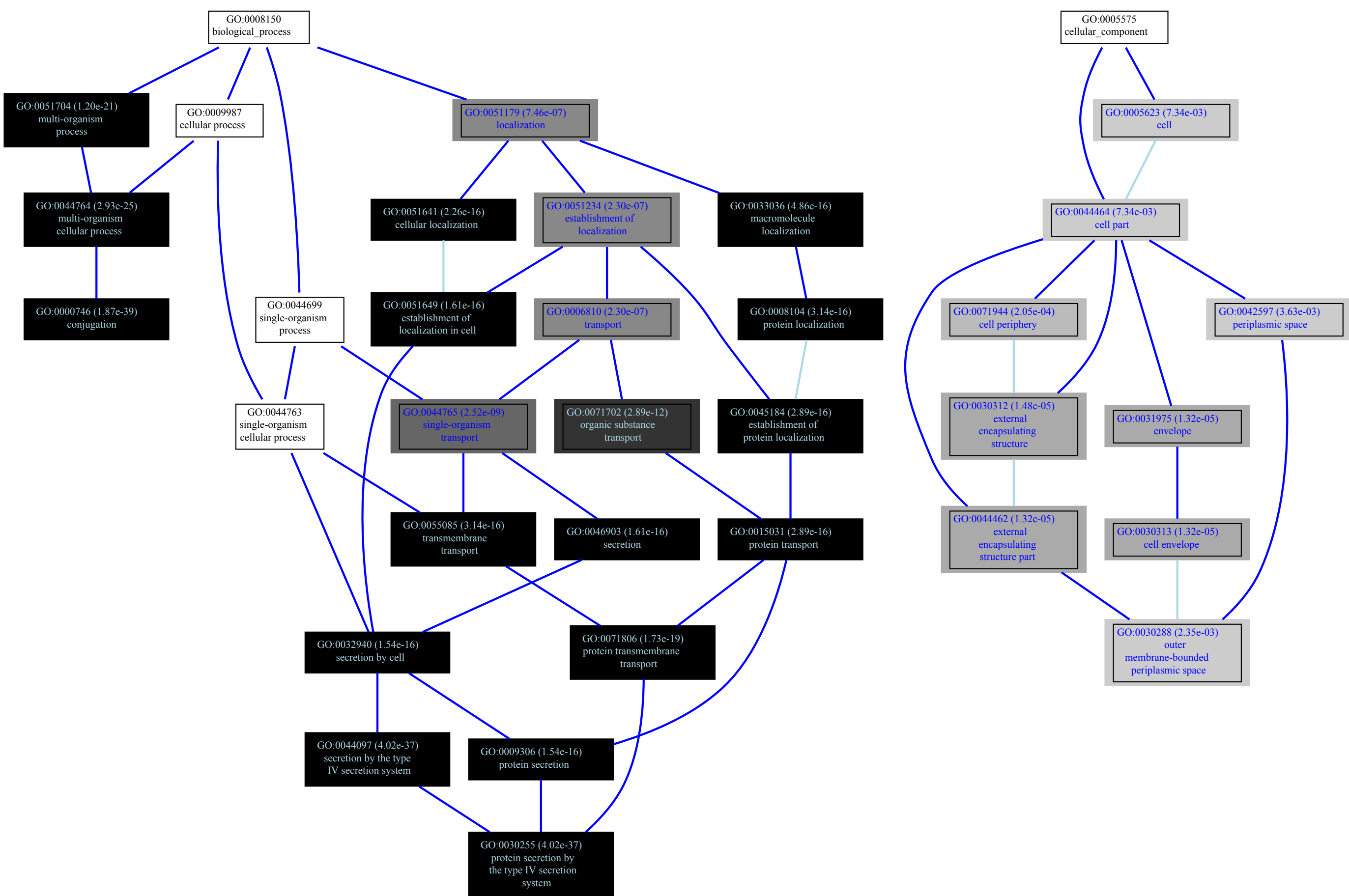

Supplement: S2 Fig — Shading corresponds to level of significance. The vertical axis generally indicates greater or lesser specificity of the GO term because of the hierarchical definitions of the terms. The three major biological functions that are enriched are those of gene expression and translation, nucleotide and ribonucelotide biosynthesis, and respiration. The majority of unboxed terms are mostly general terms lacking a unifying biological function. (PDF) [file pone.0144514.s002.pdf]

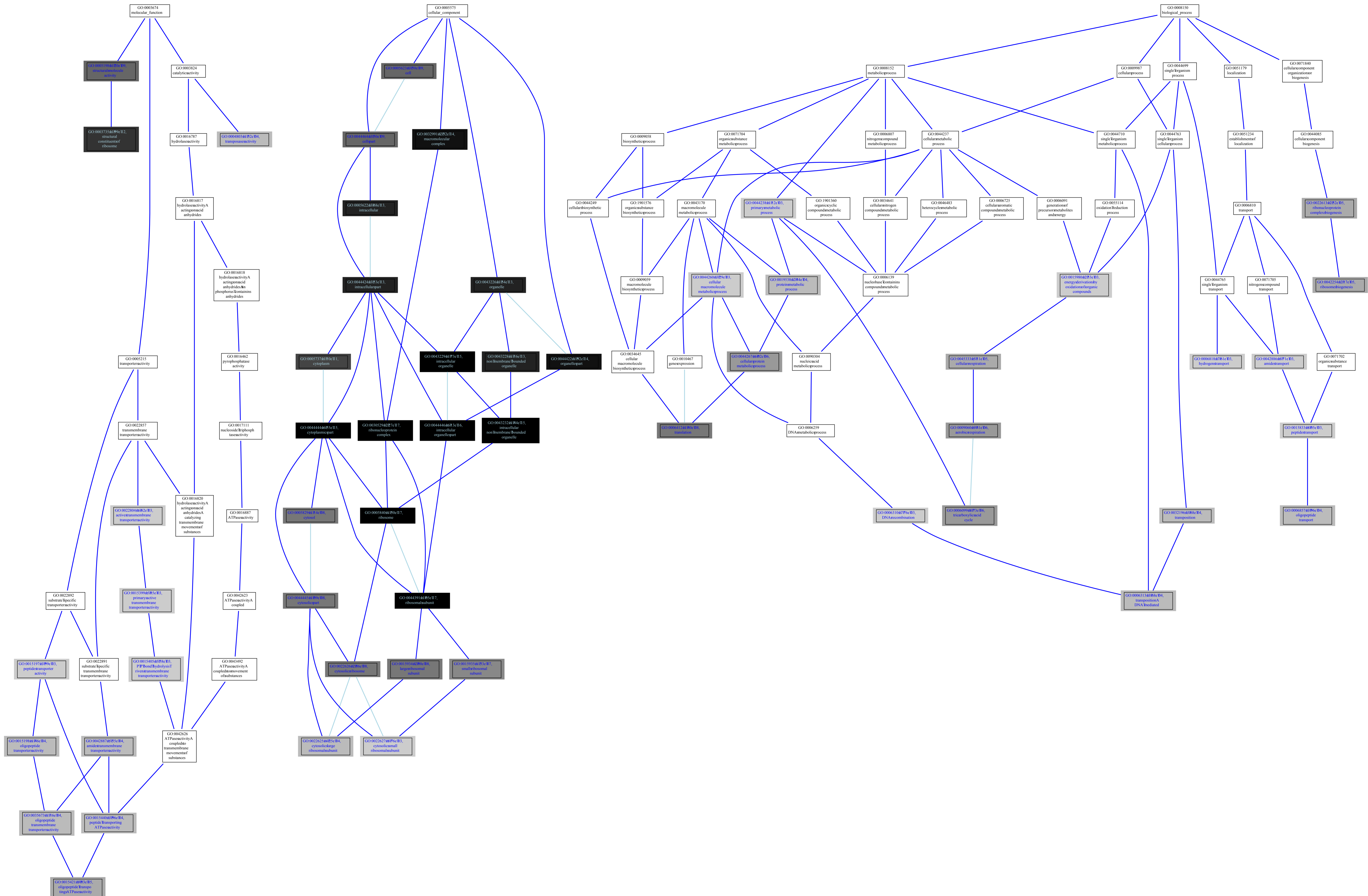

Supplement: S3 Fig — Shading corresponds to level of significance. The vertical axis generally indicates greater or lesser specificity of the GO term because of the hierarchical definitions of the terms. The three major biological functions that are enriched are those of gene expression and translation, nucleotide and ribonucelotide biosynthesis, and respiration. The majority of unboxed terms are mostly general terms lacking a unifying biological function. (PDF) [file pone.0144514.s003.pdf]

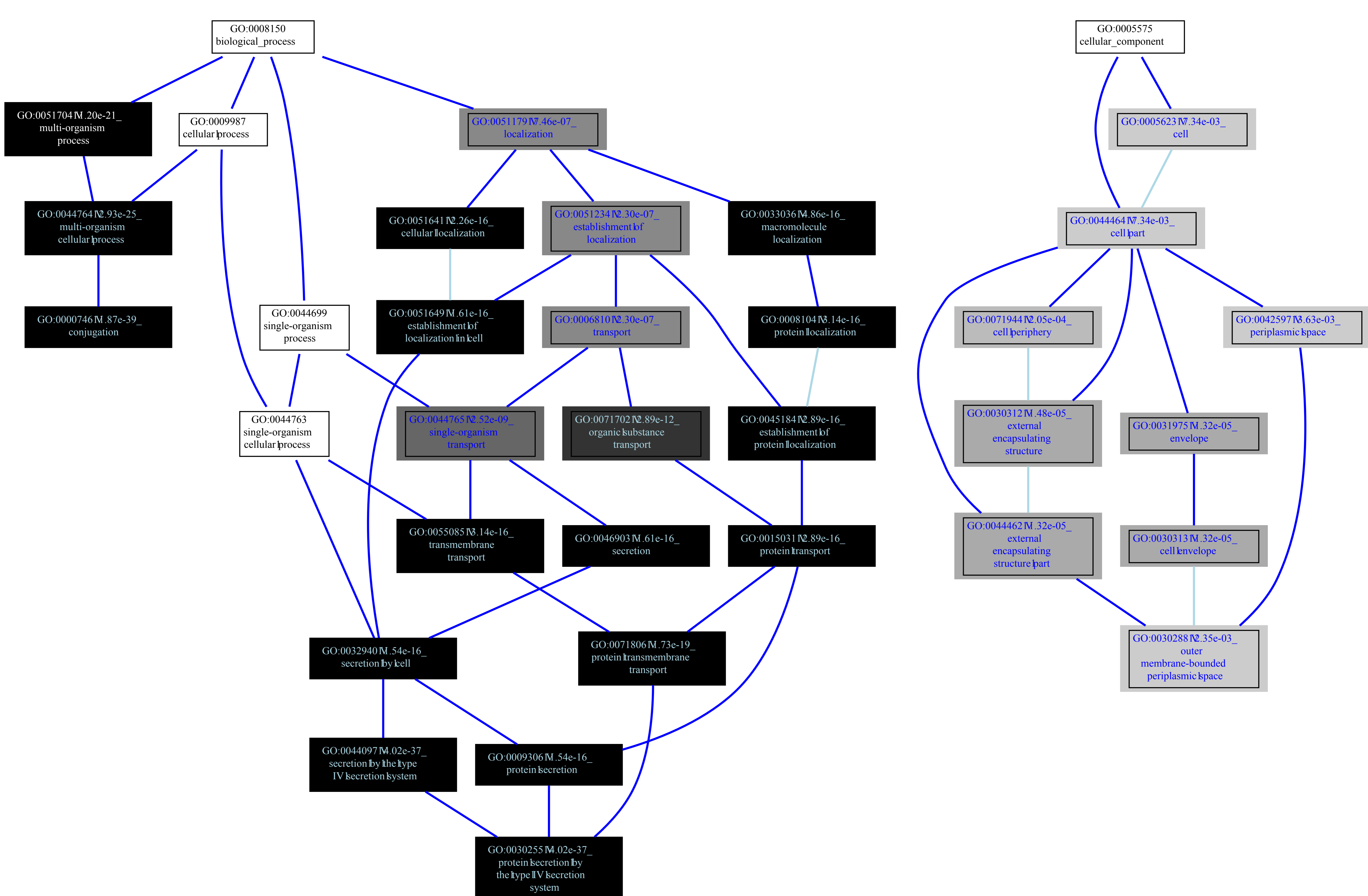

Supplement: S4 Fig — Shading corresponds to level of significance. The vertical axis generally indicates greater or lesser specificity of the GO term because of the hierarchical definitions of the terms. The three major biological functions that are enriched are those of gene expression and translation, nucleotide and ribonucelotide biosynthesis, and respiration. The majority of unboxed terms are mostly general terms lacking a unifying biological function. (PDF) [file pone.0144514.s004.pdf]
